# Astaxanthin has a beneficial influence on pain-related symptoms and opioid-induced hyperalgesia in mice with diabetic neuropathy-evidence from behavioral studies

**DOI:** 10.1007/s43440-024-00671-9

**Published:** 2024-11-12

**Authors:** Katarzyna Ciapała, Katarzyna Pawlik, Agata Ciechanowska, Wioletta Makuch, Joanna Mika

**Affiliations:** Department of Pain Pharmacology, Maj Institute of Pharmacology, Polish Academy of Sciences, Smętna 12, Kraków, 31-343 Poland

**Keywords:** Astaxanthin, Morphine, Neuropathic pain, Diabetes, Opioid-induced hyperalgesia

## Abstract

**Background:**

The treatment of painful diabetic neuropathy is still a clinical problem. The aim of this study was to determine whether astaxanthin, a substance that inhibits mitogen-activated protein kinases, activates nuclear factor erythroid 2-related factor 2 and influences N-methyl-D-aspartate receptor, affects nociceptive transmission in mice with diabetic neuropathy.

**Methods:**

The studies were performed on streptozotocin-induced mouse diabetic neuropathic pain model. Single intrathecal and intraperitoneal administrations of astaxanthin at various doses were conducted in both males and females. Additionally, repeated twice-daily treatment with astaxanthin (25 mg/kg) and morphine (30 mg/kg) were performed. Hypersensitivity was evaluated with von Frey and cold plate tests.

**Results:**

This behavioral study provides the first evidence that in a mouse model of diabetic neuropathy, single injections of astaxanthin similarly reduce tactile and thermal hypersensitivity in both male and female mice, regardless of the route of administration. Moreover, repeated administration of astaxanthin slightly delays the development of morphine tolerance and significantly suppresses the occurrence of opioid-induced hyperalgesia, although it does not affect blood glucose levels, body weight, or motor coordination. Surprisingly, astaxanthin administered repeatedly produces a better analgesic effect when administered alone than in combination with morphine, and its potency becomes even more pronounced over time.

**Conclusions:**

These behavioral results provide a basis for further evaluation of the potential use of astaxanthin in the clinical treatment of diabetic neuropathy and suggest that the multidirectional action of this substance may have positive effects on relieving neuropathic pain in diabetes.

## Highlights

In a mouse model of diabetic neuropathic pain, astaxanthin:

- attenuates pain-related behavior in male and female mice after a single administration.

- slightly delays the development of tolerance after repeated administration.

- does not disturb motor coordination and does not influence blood glucose levels.

- produces long-lasting, better than morphine, analgesia after repeated administration.

## Introduction

Diabetes mellitus is a metabolic disease that, over time, leads to serious consequences, such as blindness, heart attacks, kidney failure, stroke, or lower limb amputation. The number of people suffering from diabetes increased from 108 million in 1980 to 422 million in 2014 [1]. Importantly, chronic hyperglycemia causes nerve damage, which consequently may induce the development of neuropathic pain [2]. Even opioids, which are among the strongest analgesics, do not provide sufficient pain relief in patients with diabetic neuropathy, and their long-term use is associated with side effects [3].

The molecular basis of diabetic neuropathy is complex and still not fully known [2, 4–8]. Interestingly, it has been reported that among patients with diabetes, males develop neuropathy earlier than females and have a greater frequency of pain [9, 10], while women are more likely to have more severe neuropathic pain symptoms [11, 12]. There is an increasing need for studies to identify the most potent drugs or their combinations for the treatment of diabetic neuropathic pain to obtain a satisfactory analgesic effect and improve the quality of life of affected individuals.

It is currently believed that neuronal and immune cells play key roles in the development of neuropathic pain [13–15], and notably, their activation is closely related to the disruption of certain intracellular signaling pathways [16]. One of these cascades which regulates the nociceptive response is the mitogen-activated protein kinases (MAPK) family. This is an evolutionarily conserved group that consists of the p38 mitogen-activated protein (p38), extracellular-regulated (ERK), and c-Jun N-terminal (JNK) kinases [17–19]. A lot of previous research has indicated that MAPKs are involved in the modulation of nociceptive signals and central sensitization [20–24] and opioid effectiveness [25–29]. In recent years, a variety of selective MAPK inhibitors, e.g., SB203580, D-JNKI-1, SP600125, and PD198306, have been shown to significantly attenuate tactile and thermal hypersensitivity in animal models [23, 30, 31]. Moreover, some evidence implies that, among transcription factors, nuclear factor erythroid 2-related factor 2 (Nrf2) may be an important target for the pharmacological treatment of neuropathic pain [32]. Recent studies have indicated that a single intrathecal (*it*) injection of astaxanthin, a substance that inhibits MAPKs and activates Nrf2 [33–36], provides pain relief and improves opioid effectiveness in male mice exposed to sciatic nerve injury [37]. Moreover, it has recently been suggested that this substance also acts through N-methyl-D-aspartate (NMDA) receptors, which are very important for pain processes [38, 39]. Astaxanthin is a carotenoid primarily present in marine organisms [40]. It has gained significant attention for its therapeutic advantages in conditions related to various diseases via its potential to improve oxidative stress, inflammation, and lipid/glucose metabolism [40, 41]. Recently, PeaNoc XL (containing 1 mg of astaxanthin) has been shown to reduce pain and joint stiffness in patients with arthritis [42].

Therefore, in light of the lack of knowledge regarding the usefulness of astaxanthin in the treatment of diabetic neuropathy, the goal of our first behavioral experiments was to determine the effects of single *it* and intraperitoneal (*ip*) injections of this substance on tactile and thermal hypersensitivity in male and female mice with streptozotocin (STZ)-induced diabetic neuropathic pain. Furthermore, the second behavioral study performed only in male mice was aimed to evaluate whether and how repeated *ip* injections of astaxanthin influence pain relief, morphine tolerance development, glucose levels, body weight, and motor coordination in a model of diabetic neuropathic pain.

## Materials and methods

### Animals

Male and female albino Swiss mice (20–22 g) were acquired from Charles River (Hamburg, Germany) and held in cages with sawdust on a standard 12 h/12 h light/dark cycle (lights on at 06:00 a.m.), with food and water available *ad libitum*. All of the procedures were carried out in agreement with the recommendations of the International Association for the Study of Pain [43] and the National Institutes of Health Guide for the Care and Use of Laboratory Animals and were approved by the II Local Ethics Committee of the Maj Institute of Pharmacology, Polish Academy of Sciences (LKE 252/2023; 128/2023; 124/2024). Care was taken to minimize animal suffering and reduce the number of animals used in the experiments (3R policy). The total number of animals in the following study is 185.

### Diabetic neuropathic pain model

Streptozotocin (STZ) is one of the chemical components most commonly used in experimental diabetes research [44–47] since it causes specific necrosis of the pancreatic beta cells [48, 49]. In our experiments, a single intraperitoneal (*ip*) injection of STZ (200 mg/kg; Sigma Aldrich, St. Louis, USA), dissolved in water, was used to establish a diabetes type 1 model in 5–6 weeks old mice [27]. As a control group, age-matched nondiabetic (naive) mice received water instead. Blood was collected from the tail veins of the mice, and the glucose concentration was measured with an Accu-Chek Active glucometer (Warsaw, Poland). The measurement range of the glucometer ends at 600 mg/dL. The mice were considered to have developed diabetes when their glucose level was greater than 300 mg/dL between days 5 and 7 after STZ injection. On days 5–7 after surgery, the majority of STZ-exposed mice fully developed tactile and thermal hypersensitivity compared to that of naive animals, as measured by the von Frey and cold plate tests. Mice were randomly selected for the experimental groups. After administering STZ, we observed that the average glucose level in males was 569 and that in females was 580 mg/dL.

### Pharmacological study

The following compounds were used in our experiments: astaxanthin (A; MedChemExpress, Monmouth Junction, NJ, USA) and morphine hydrochloride (M; Fagron, Krakow, Poland). Substances were administered by intrathecal (*it*) or *ip* injection. Astaxanthin solutions were red-purple, while the vehicles were transparent. The *it* route of administration is a standard procedure in our laboratory [37, 50]. It is achieved by using a Hamilton syringe with a thin needle in accordance with the guidelines described previously [51]. The injections were performed in the lumbar segment of the spinal cord (between the L5 and L6 vertebrae) with a volume of 5 µl, and the tail reflex was used as an indicator of proper administration.

#### Dose-dependency study

Astaxanthin was administered *it* at doses of 0.5, 1, 2, or 4 µg/5 µl or *ip* at doses of 1, 10, 25, or 50 mg/kg on day 7 after STZ treatment. These doses were selected based on our previous studies and the available literature [37, 52]. The behavioral tests were conducted 0.5, 1.5, 3, 5, and 24 h after these injections. The control groups for *it* administration were injected with 60% DMSO (5 µl) at the same time points, while the control groups for *ip* administration received 30% propylene glycol.

#### Repeated coadministration of astaxanthin with morphine

In STZ-treated male mice, tolerance development and opioid-induced hyperalgesia were studied by administering morphine twice daily at the dose of 30 mg/kg, *ip* (similarly as described by others [53, 54]) with or without astaxanthin (twice daily 25 mg/kg, *ip*) for 17 days, starting from the 5th day after STZ injection. In the experimental groups, astaxanthin was administered twice daily, 30 min before each morphine injection, for the following 17 days. The behavioral tests were conducted 30 min after morning morphine administration, in the initial phase (up to day 7) every second day and later, due to the rapidly developing morphine tolerance, daily. Additionally, on days 9 and 17, after behavioral tests blood glucose concentrations, motor coordination, and body weight measurements were performed.

### Behavioral tests

#### Von Frey test

Mechanical hypersensitivity was measured using calibrated nylon monofilaments of increasing strength (0.6 to 6 g; 6 g is the cutoff latency) (Ugo Basile, Gemonio, Italy) to observe reactions to tactile stimuli, as described previously [55]. The mice were habituated in plastic cages with a wire mesh floor 5 min before the experiment, and von Frey filaments were applied to the midplantar surface of the hind paws until the limbs were lifted. This test is used regularly in our laboratory [56, 57].

#### Cold plate test

Thermal hypersensitivity was measured using a cold plate analgesia meter (Ugo Basile, Gemonio, Italy) as described previously [58]. The temperature of the cold plate was maintained at 2 °C, and the cutoff latency was 30 s. The mice were positioned on the cold plate separately, and the latency to lift the hind paw was noted. Both hind paws in naive and STZ-exposed mice were observed simultaneously. This test is used regularly in our laboratory [56, 57].

#### Rotarod test

The rotarod test is a frequently used method to assess motor coordination in animals and was used in our previous studies [59]. The mice were placed in separate compartments on a horizontal rod that rotated at an accelerating speed, starting at 2 rpm and reaching 40 rpm within 300 s. The animals were acclimated to the apparatus and trained on the rotating rod. The time until the mouse fell off the rod was recorded. The rotarod test was conducted as a part of a repeated administration study, 1 h after morphine administration on days 9 and 17. The cutoff latency was 300 s.

### Data analysis

**Fig. 1:**
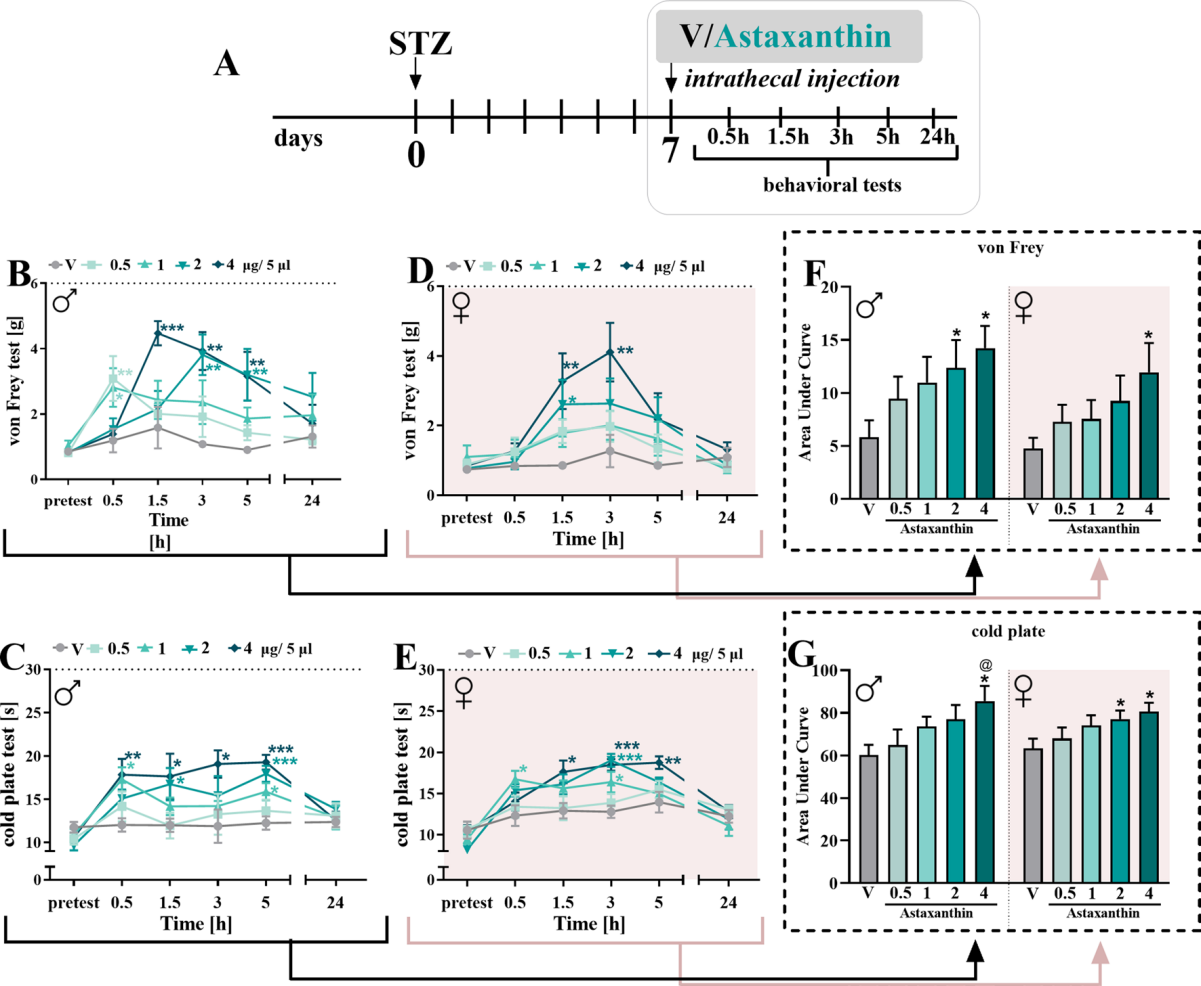
Influence of *it* astaxanthin administration on pain-related behavior in STZ-treated male and female mice **(A)**. The effects of injections of astaxanthin (0.5, 1, 2 and 4 µg/5µL) on tactile and thermal hypersensitivity were assessed at 0.5, 1.5, 3, 5 and 24 h after treatment on the 7th day post-STZ in males **(B**,** C)** and females **(D**,** E).** The data are presented as the means ± SEMs and the numbers of animals are as follow: males: V = 7–8; 0.5 µg/5µL = 7–8; 1 µg/5µL = 8; 2 µg/5µL = 8; 4 µg/5µL = 7–8 and females: V = 7; 0.5 µg/5µL = 7–8; 1 µg/5µL = 6–8; 2 µg/5µL = 8; 4 µg/5µL = 6–8. Additionally, the data obtained from the von Frey (**B**,**D**) and cold plate (**C**,**E**) tests were analyzed as areas under the curve (**F**,**G**) to visualize overall changes between doses efficacy. The intergroup differences were analyzed using one-way ANOVA with Bonferroni’s multiple comparisons test. * *p* < 0.05, ** *p* < 0.01 and *** *p* < 0.001 indicate differences between V-treated STZ-exposed mice and substance-treated STZ-exposed mice; @ *p* < 0.05 indicates differences between animals treated with astaxanthin at the dose of 0.5 µg/5 µL. Moreover, treatment x time points interaction were measured using two-way repeated measures ANOVA. The dotted lines indicate the cutoff values for the tests

**Fig. 2:**
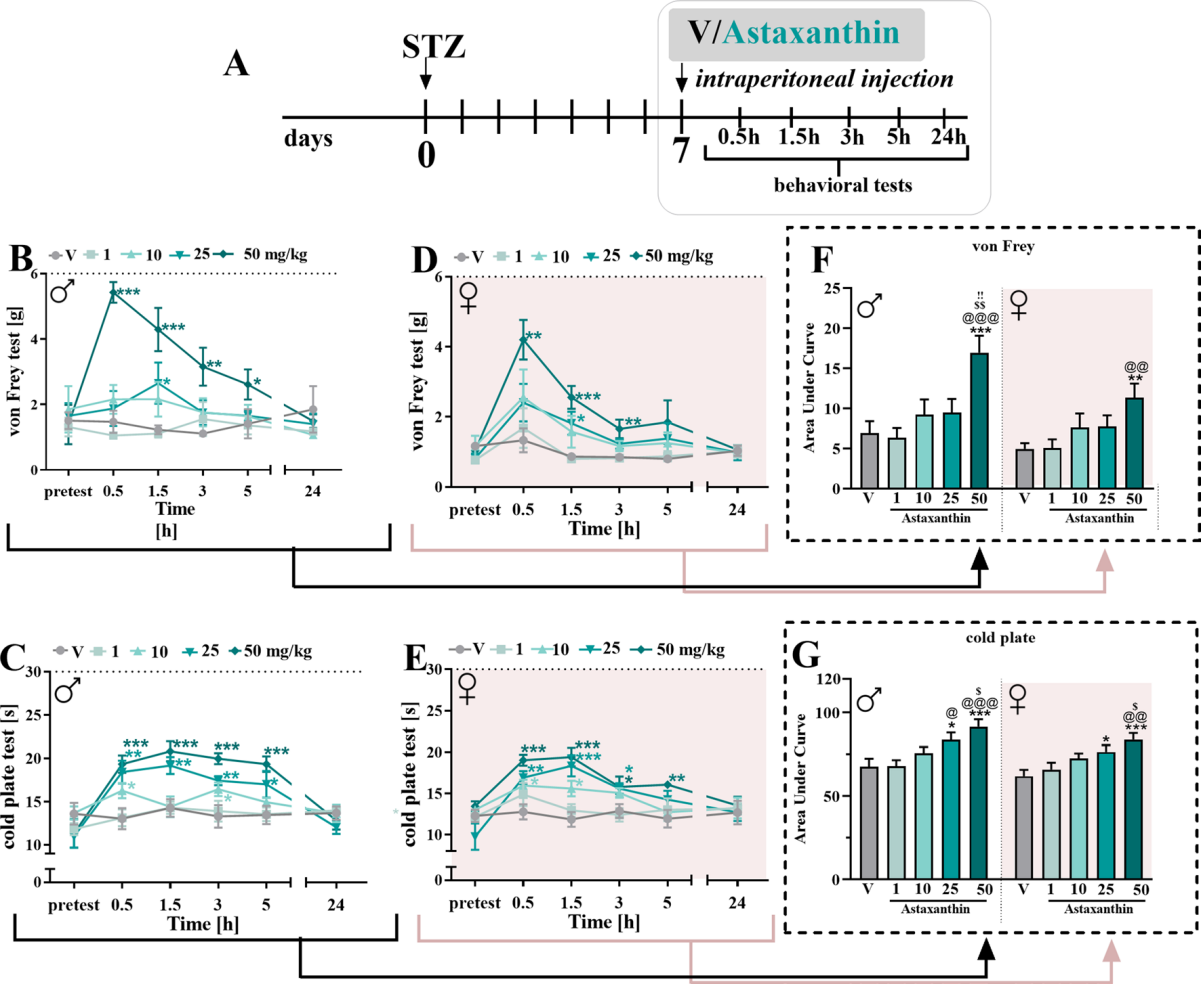
Influence of *ip* astaxanthin administration on pain-related behavior in STZ-treated male and female mice **(A)**. The effects of injections of astaxanthin (1, 10, 25 and 50 mg/kg) on tactile and thermal hypersensitivity were assessed at 0.5, 1.5, 3, 5 and 24 h after treatment on the 7th day post-STZ in males (**B**,** C**) and females (**D**,** E**). The data are presented as the means ± SEMs and the numbers of animals are as follow: males: V = 7; 1 mg/kg = 7; 10 mg/kg = 7; 25 mg/kg = 6; 50 mg/kg = 7 and females: V = 6; 1 mg/kg = 6; 10 mg/kg = 6; 25 mg/kg = 6; 50 mg/kg = 7. Additionally, the results obtained from the von Frey (**B**,**D**) and cold plate (**C**,**E**) tests were analyzed as areas under the curve (**F**,**G**) to visualize overall changes between doses efficacy. The intergroup differences were analyzed using one-way ANOVA with Bonferroni’s multiple comparisons test. * *p* < 0.05, ** *p* < 0.01 and *** *p* < 0.001 indicate differences between V-treated STZ-exposed mice and substance-treated STZ-exposed mice; @ *p* < 0.05, @@ *p* < 0.01 and @@@ *p* < 0.001 indicate differences between animals treated with astaxanthin at the dose of 1 mg/kg; $ *p* < 0.05, $$ *p* < 0.01 indicate differences between animals treated with astaxanthin at the dose of 10 mg/kg; !! *p* < 0.01 indicates differences between animals treated with astaxanthin at the dose of 25 mg/kg 0.01. Moreover, treatment x time points interaction were measured using two-way repeated measures ANOVA. The dotted lines indicate the cutoff values for the tests

**Fig. 3:**
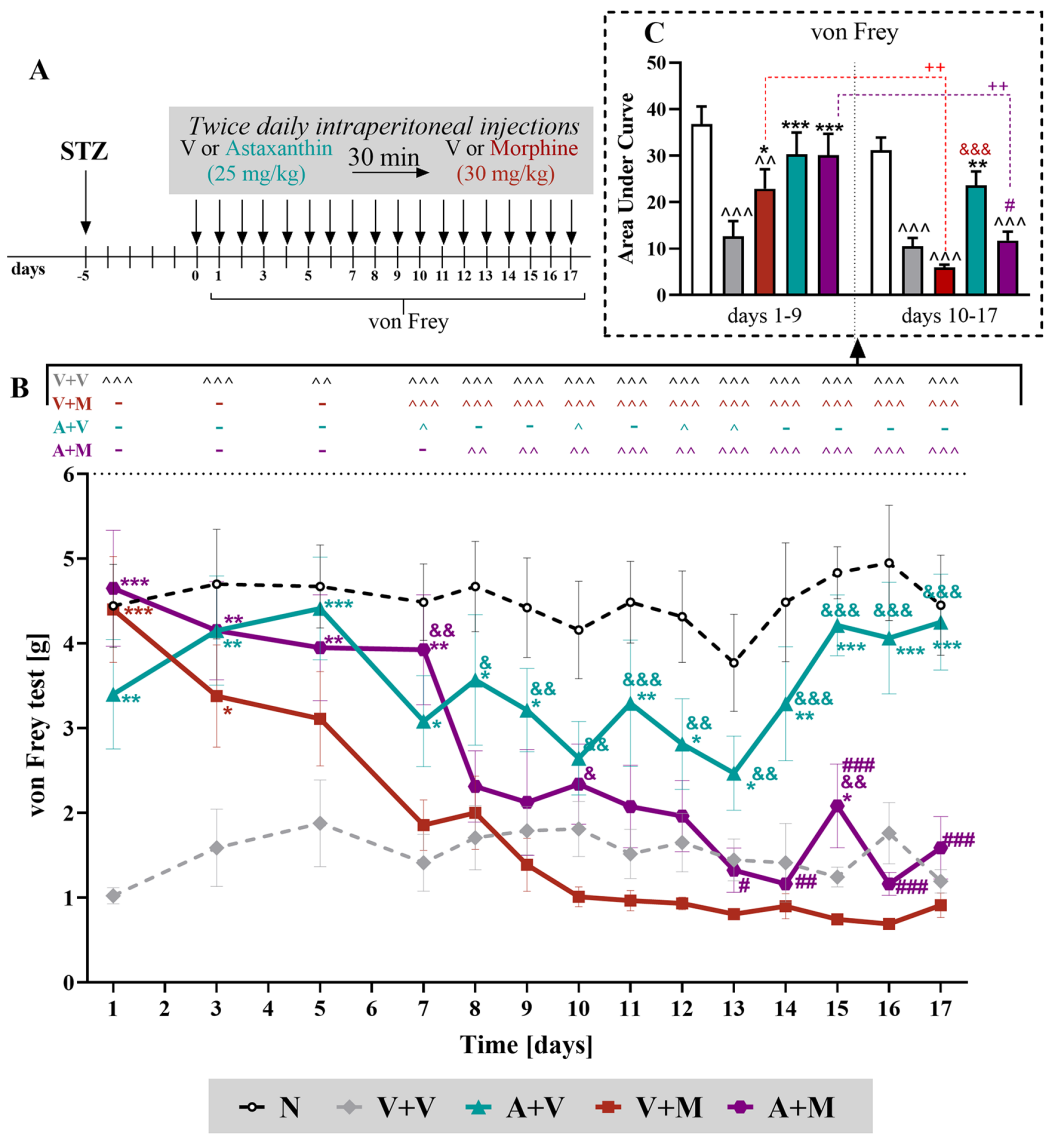
Repeated twice daily *ip* administration of astaxanthin (25 mg/kg) influenced morphine (30 mg/kg) tolerance as measured by von Frey in male mice (**A**). V and astaxanthin (25 mg/kg) were repeatedly administered on the 5th day after STZ injection and then twice daily for 17 days, 30 min before treatment with V or morphine (M, 30 mg/kg); 30 min later, the von Frey (**B**) tests were conducted. The data are presented as the mean ± SEM, and the numbers of animals are as follows: *N* = 6–7; V + V = 9; V + M = 9; A + V = 8; A + M = 8. The results were analyzed using one-way ANOVA with Bonferroni’s multiple comparisons *post hoc* test. Additionally, the data obtained from the von Frey **(B)** test were analyzed as areas under the curve **(C)** in late and early phase of experiment to visualize overall changes between doses efficacy. ^ *p* < 0.05, ^^ *p* < 0.01, ^^^ *p* < 0.001 indicate differences between naive and STZ-exposed mice; * *p* < 0.05, ** *p* < 0.01, and *** *p* < 0.001 indicate differences compared with V + V-treated STZ-exposed mice; & *p* < 0.05, && *p* < 0.01 and &&& *p* < 0.001 indicate differences compared with the V + M-treated STZ-exposed mice; # *p* < 0.05, ## *p* < 0.01 and ### *p* < 0.001 indicate differences compared to the A + V-treated STZ-exposed mice. Furthermore, the t test was used to compare the two time intervals from AUC analysis (**C**) and + + *p* < 0.01 indicates differences between the respective groups. Moreover, treatment x time points interaction were measured using two-way repeated measures ANOVA. The dotted lines show the cutoff values for the tests. Abbreviations: A, astaxanthin; M, morphine; N, naive; V, vehicle; STZ, streptozotocin

**Fig. 4:**
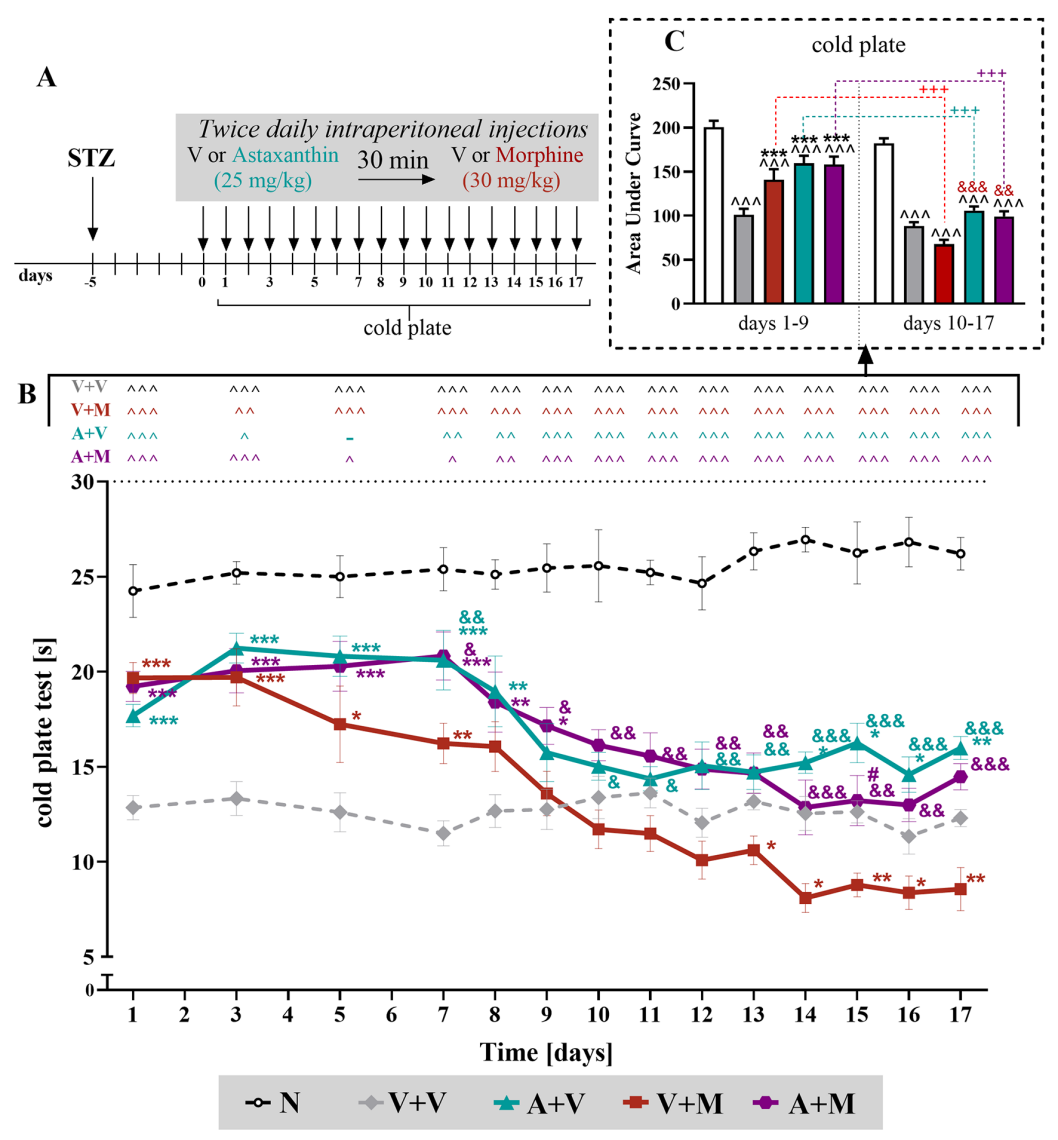
Repeated twice daily *ip* administration of astaxanthin (25 mg/kg) influenced morphine (30 mg/kg) tolerance and opioid-induced hyperalgesia, as measured by cold plate in male mice **(A)**. V and astaxanthin (25 mg/kg) were repeatedly administered on the 5th day after STZ injection and then twice daily for 17 days, 30 min before treatment with V or morphine (M, 30 mg/kg); 35 min later cold plate (**B**) tests were conducted. The data are presented as the mean ± SEM, and the numbers of animals are as follows: *N* = 6–7; V + V = 9; V + M = 9; A + V = 8; A + M = 8. The results were analyzed using one-way ANOVA with Bonferroni’s multiple comparisons *post hoc* test. Additionally, the data obtained from the cold plate **(B)** test were analyzed as areas under the curve **(C)**, in late and early phase of experiment to visualize overall changes between doses efficacy. ^ *p* < 0.05, ^^ *p* < 0.01, ^^^ *p* < 0.001 indicate differences between naive and STZ-exposed mice; * *p* < 0.05, ** *p* < 0.01, and *** *p* < 0.001 indicate differences compared with V + V-treated STZ-exposed mice; & *p* < 0.05, && *p* < 0.01 and &&& *p* < 0.001 indicate differences compared with the V + M-treated STZ-exposed mice; # *p* < 0.05, indicates differences compared to the A + V-treated STZ-exposed mice. Furthermore, the t test was used to compare the two time intervals from AUC analysis (**C**) and +++ *p* < 0.01 indicates differences between the respective groups. Moreover, treatment x time points interaction were measured using two-way repeated measures ANOVA. The dotted lines show the cutoff values for the tests. Abbreviations: A, astaxanthin; M, morphine; N, naive; V, vehicle; STZ, streptozotocin

The Shapiro-Wilk test was performed to evaluate the normality of variable distributions (significance level α = 0.01). The behavioral data are presented as the means ± SEM in grams or seconds. One-way analysis of variance (ANOVA) was used to evaluate the experimental results, followed by the Bonferroni post hoc test of selected pairs, measured separately at each time point. The results shown in Figs. 1, 2 and 3, and 4 were additionally evaluated using two-way repeated measures ANOVA to detect time–drug interactions, and in the case of Figs. 3 and 4 also to investigate the occurrence of opioid-induced hyperalgesia. In addition, we have added the area under the curve (AUC) results as an inserts to visualize efficacy of each dose between sexes (Figs. 1 and 2) and to compare the effectiveness of the each treatment in two intervals (1–9 days and 10–17 days) in Figs. 3 and 4 - this data were evaluated using t-test. The AUC was calculated by trapezoidal and Simpson’s rules, as described by Tallarida and Murray [60]. The Litchfield and Wilcoxon method was applied to determine the antinociceptive dose necessary to produce a 50% response (ED_50_) and the 95% confidence intervals of the quantitative data, which were automatically calculated with Pharm/PCS software (version 4) for the results obtained 1.5 h after drug administration. In the experiment with chronic astaxanthin and morphine administration, the area under the curve was calculated to compare the effects of the tested substances between selected time intervals. All of the statistical analyses and graphs were performed using Prism (version 9.1.2 (226), GraphPad Software, Inc., San Diego, CA, USA). Differences were considered significant when *p* < 0.05.

## Results

### Effect of a single administration of astaxanthin on tactile and thermal hypersensitivity on the 7th day after STZ treatment in male and female mice

Single *it* administrations of astaxanthin at different doses (0.5, 1, 2, and 4 µg/5 µl) were performed on day 7 after STZ injection in male and female mice (Fig. 1A), and the influence of the drug on hypersensitivity to tactile (Fig. 1B, D) and thermal (Fig. 1C, E) stimuli was measured.

According to the von Frey test, astaxanthin reduced mechanical hypersensitivity in both sexes. In males, the most effective dose of astaxanthin was 4 µg/5 µl, which had the greatest effect on mechanical hypersensitivity at 1.5 h (F_4,35_=4.753; *p* = 0.0036) after injection; however, this effect was also significant after 3 h (Fig. 1B). A dose of 2 µg/5 µl had an analgesic effect 3 h after administration (F_4,34_=4.788; *p* = 0.0036), while doses of 0.5 and 1 µg/5 µl reduced mechanical hypersensitivity only 0.5 h after injection (F_4,35_=3.562; *p* = 0.0154). Analgesic effects were no longer observed 24 h after administration (Fig. 1B). Two-way repeated measures ANOVA confirmed a significant interaction between the investigated treatment and the tested time points (F_20,206_=2.732; *p* = 0.0002). Similarly, astaxanthin at the highest dose of 4 µg/5 µl significantly reduced mechanical hypersensitivity at 1.5 (F_4,34_=2.713; *p* = 0.0461) and 3 h (F_4,34_=3.061; *p* = 0.0295) after administration in female mice (Fig. 1D). A dose of 2 µg/5 µl had an analgesic effect only 1.5 h after injection (Fig. 1D). Two-way repeated measures ANOVA did not confirm a significant interaction between the investigated treatment and the tested time points. Moreover, analysis of the area under the curve, which was performed for each dose used, revealed that after *it* administration, astaxanthin had a similar effect on hypersensitivity to mechanical stimuli in both male and female mice with diabetic neuropathic pain (Fig. 1F).

According to the cold plate test, astaxanthin reduced thermal hypersensitivity in both genders. In males, the best analgesic effects were observed at a dose of 4 µg/5 µl 5 h after administration (F_4,33_=8.783; *p* < 0.0001); however, this effect was also significant 1.5 and 3 h after injection (Fig. 1C). A dose of 2 µg/5 µl had an analgesic effect only 5 h after administration, while doses of 0.5 and 1 µg/5 µl did not reduce thermal hypersensitivity at any tested time point (Fig. 1C). Two-way repeated measures ANOVA did not confirm a significant interaction between the investigated treatment and the subjected time point. Similarly, astaxanthin at the highest dose of 4 µg/5 µl evoked the most significant reduction in thermal hypersensitivity at 3 h (F_4,33_=7.937; *p* = 0.0001) after administration in female mice; however, this effect was also significant at 5 h after injection (F_4,33_=3.135; *p* = 0.0273) (Fig. 1E). The doses of 1 and 2 µg/5 µl had analgesic effects only 3 h after injection (Fig. 1E). Analgesic effects were no longer observed 24 h after administration. Two-way repeated measures ANOVA confirmed a significant interaction between the treatment and the tested time points (F_20,196_=2.323; *p* = 0.0017). Moreover, analysis of the area under the curve in the cold plate test showed that after *it* administration, astaxanthin had a similar effect on hypersensitivity to thermal stimuli in both male and female mice with diabetic neuropathic pain (Fig. 1G).

### The effect of a single *ip* administration of astaxanthin on tactile and thermal hypersensitivity on the 7th day after STZ treatment in male and female mice

Single *ip* injections of astaxanthin at different doses (1, 10, 25, and 50 mg/kg) were performed on day 7 after STZ administration in male and female mice (Fig. 2A), and the influence of the drug on hypersensitivity to tactile (Fig. 2B, D) and thermal (Fig. 2C, E) stimuli was measured.

According to the von Frey test, astaxanthin reduced mechanical hypersensitivity in both sexes. In male mice, the most effective dose of this substance was 50 mg/kg, which had the greatest effect on mechanical hypersensitivity at 0.5 h (F_4,29_=23.47; *p* < 0.0001); however, this dose was also effective at 1.5 h and 3 h after injection (Fig. 2B). A dose of 25 mg/kg showed an analgesic effect 1.5 h after administration (F_4,29_=7.772; *p* = 0.0002), while the other doses of 1 and 10 mg/kg did not cause a decrease in mechanical hypersensitivity at any time point after treatment. Analgesic effects were no longer observed 24 h after administration (Fig. 2B). Two-way repeated measures ANOVA confirmed a significant interaction between the investigated treatment and the tested time points (F_20,174_=3.145; *p* < 0.0001). Similarly, at the highest dose of 50 mg/kg, astaxanthin caused the greatest reduction in mechanical hypersensitivity at 0.5 h (F_4,26_=3.879; *p* = 0.0134) after administration, but it was also effective at 1.5 h (F_4,26_=6.091; *p* = 0.0013) and 3 h (F_4,26_=4.106; *p* = 0.0104) post-treatment in female mice (Fig. 2D). The dose of 25 mg/kg decreased mechanical hypersensitivity only 1.5 h after injection. The other doses did not cause a decrease in mechanical hypersensitivity at any time point after treatment. Two-way repeated measures ANOVA confirmed a significant interaction between the investigated treatment and the tested time points (F_20,156_=1.799; *p* = 0.0249). Moreover, the analysis of the area under the curve revealed that each dose of astaxanthin after *ip* administration had a similar effect on hypersensitivity to mechanical stimuli in both male and female mice with diabetic neuropathic pain (Fig. 2F).

According to the cold plate test results, astaxanthin reduced thermal hypersensitivity in both sexes. In males, the best analgesic effects were observed at a dose of 50 mg/kg for 1.5 h (F_4,29_=9.351; *p* < 0.0001); however, this dose was also effective at 0.5 h, 3 h and 5 h after administration (Fig. 2C). A dose of 25 mg/kg had analgesic effects at 0.5 h, 1.5 h and 3 h after administration, while a dose of 10 mg/kg reduced thermal hypersensitivity at 0.5 h and 3 h after treatment. A dose of 1 mg/kg did not influence thermal hypersensitivity in diabetic males. Two-way repeated measures ANOVA confirmed a significant interaction between the investigated treatment and the tested time points (F_20,174_=3.623; *p* < 0.0001). Similarly, the most effective dose in females was 50 mg/kg, which significantly reduced thermal hypersensitivity 1.5 h (F_4,26_=10.20; *p* < 0.0001) after administration; however, it was also effective at 0.5 h, 3 h and 5 h after treatment (Fig. 2E). A dose of 25 mg/kg had analgesic effects at 0.5 h, 1.5 h and 3 h after administration, while a dose of 10 mg/kg reduced thermal hypersensitivity at 0.5 and 1.5 h after treatment. The dose of 1 mg/kg did not have any effect. Two-way repeated measures ANOVA confirmed a significant interaction between the investigated treatment and the tested time points (F_20,156_=2.208; *p* = 0.0037). Moreover, analysis of the area under the curve showed that after *ip* administration, astaxanthin had a similar effect on hypersensitivity to thermal stimuli in both male and female mice with diabetic neuropathic pain (Fig. 2G).

As specified by the calculated ED_50_ for *it* administration, the lowest value in the von Frey test was obtained for males; however, this difference was not statistically significant compared to that for females. In the case of the cold plate test, the effective *it* dose in both males and females was similar. According to *ip* administration, in both the von Frey and cold plate tests, lower ED_50_ values were calculated for males (Table 1), but these differences were not statistically significant compared with those for females. Due to the slightly better effect of astaxanthin after *ip* administration in males, experiments with repeated administration of this substance were carried out in this group, also bearing in mind that diabetic neuropathic pain develops faster in males.

**Table 1:** The ED_50_ values for the effects of *it* (0.5, 2, 4 µg/5 µl) and *ip* (1, 10, or 50 mg/kg) astaxanthin administered to male and female mice were calculated, with 95% confidence intervals for STZ-exposed mice as measured by Von Frey and cold plate tests 1.5 h after drug injection

ASTAXANTHIN			
ROUTE OF ADMINISTRATION	BEHAVIORAL TESTS	ED_50_	
		♂	♀
*it*	von Frey	1.23 (0.51–2.96)	3.01 (0.33–27.73)
	cold plate	1.48 (0.42–5.14)	1.16 (0.22–6.06)
*ip*	von Frey	20.01 (2.05-195.68)	106.52 (9.50-1194.80)
	cold plate	3.87 (0.36–41.60)	6.53 (0.93–45.97)

### The effect of repeated *ip* administration of astaxanthin on the analgesic effects of chronic treatment with morphine in STZ-exposed male mice

The influence of astaxanthin on pain-related behavior in male mice after treatment with STZ was evaluated by performing repeated injections of morphine (30 mg/kg *ip*) twice daily with or without astaxanthin (25 mg/kg, *ip*) for 17 days, starting from the 5th day after STZ administration (Figs. 3A and 4A). The results indicated that vehicle-treated STZ-treated mice exhibited mechanical and thermal hypersensitivity for 17 days. According to the von Frey test, morphine began to lose its analgesic potential beginning on the 5th day after administration, which resulted in the potentiation of tactile hypersensitivity on day 10 (Fig. 3B). Animals receiving astaxanthin showed a decrease in hypersensitivity to tactile stimuli throughout the experiment, remaining at a high level on day 17 (F_4,35_=20.32; *p* < 0.0001). Interestingly, the combined administration of astaxanthin and morphine had an analgesic effect until day 7, after which the effect started to decrease. These results indicate that astaxanthin only slightly delayed the development of morphine tolerance in the von Frey test in mice with diabetic neuropathy. Moreover, two-way repeated measures ANOVA revealed significant interactions between treatment and all time points (F_52,__501_ = 2,371; *p* < 0.0001), in the response to tactile stimuli and additionally, between vehicle- and morphine-treated mice over a period of 10–17 days indicated that morphine caused even greater sensitivity (F_4__,313_=102.0; *p* < 0.0001), which confirmed the development of opioid-induced hyperalgesia (OIH). The comparison of this response between the mice receiving morphine and the animals treated with astaxanthin and morphine showed that astaxanthin prevented the development of OIH (*p* = 0.0002). Additionally, the analysis of the area under the curve of the von Frey test (Fig. 3C), calculated for two selected intervals (days 1–9 and days 10–17, when a greater sensitivity of animals receiving morphine was observed), also confirmed that morphine significantly lost its effectiveness over time (t = 3.969; *p* = 0.0041). The effectiveness of the administration of astaxanthin combined with morphine decreased between these intervals (t = 3.690; *p* = 0.0061). However, the analgesic effect of astaxanthin remained at a similar level in the von Frey test (Fig. 3C).

The cold plate test results revealed that morphine lost its analgesic potential on the 8th day after administration, and starting from the 13th day, it did not reduce hypersensitivity to thermal stimuli but even strengthened this reaction (Fig. 4B). Repeated administration of astaxanthin did not cause a decrease in hypersensitivity to thermal stimuli only between the 9th and 13th days of administration, while at the other investigated time points, an analgesic effect was observed. Interestingly, the combined administration of astaxanthin and morphine reduced thermal hypersensitivity until day 9, and this coadministration had better effects than morphine until the end of the experiment. These results indicate that astaxanthin slightly delay the development of morphine tolerance in the cold plate test in animals with diabetic neuropathy; however, it prevented OIH. This finding was also proved by two-way repeated measures ANOVA analysis of the animal’s response to thermal stimuli over a period of 10–17 days, which indicated that morphine caused even greater sensitivity (F_4,313_=291.7; *p* < 0.0001), which confirmed the development of OIH. We also noted a decrease in hypersensitivity in mice treated with astaxanthin and morphine in comparison to that in mice treated with morphine alone (*p* < 0.0001). However, significant interaction between treatment and all time points were confirmed using two-way repeated measures ANOVA (F_52, 501_ = 3,884). Calculation of the area under the curve for the cold plate test also confirmed that morphine lost its effectiveness (t = 5.647; *p* = 0.0005), but this effect was observed as well for astaxanthin (t = 5.493; *p* = 0.0006) and for their coadministration (t = 5.435; *p* = 0.0006) (Fig. 4C).

### The influence of repeated *ip* administration of astaxanthin and morphine on motor coordination, blood glucose levels and body weight in STZ-treated male mice

**Fig. 5:**
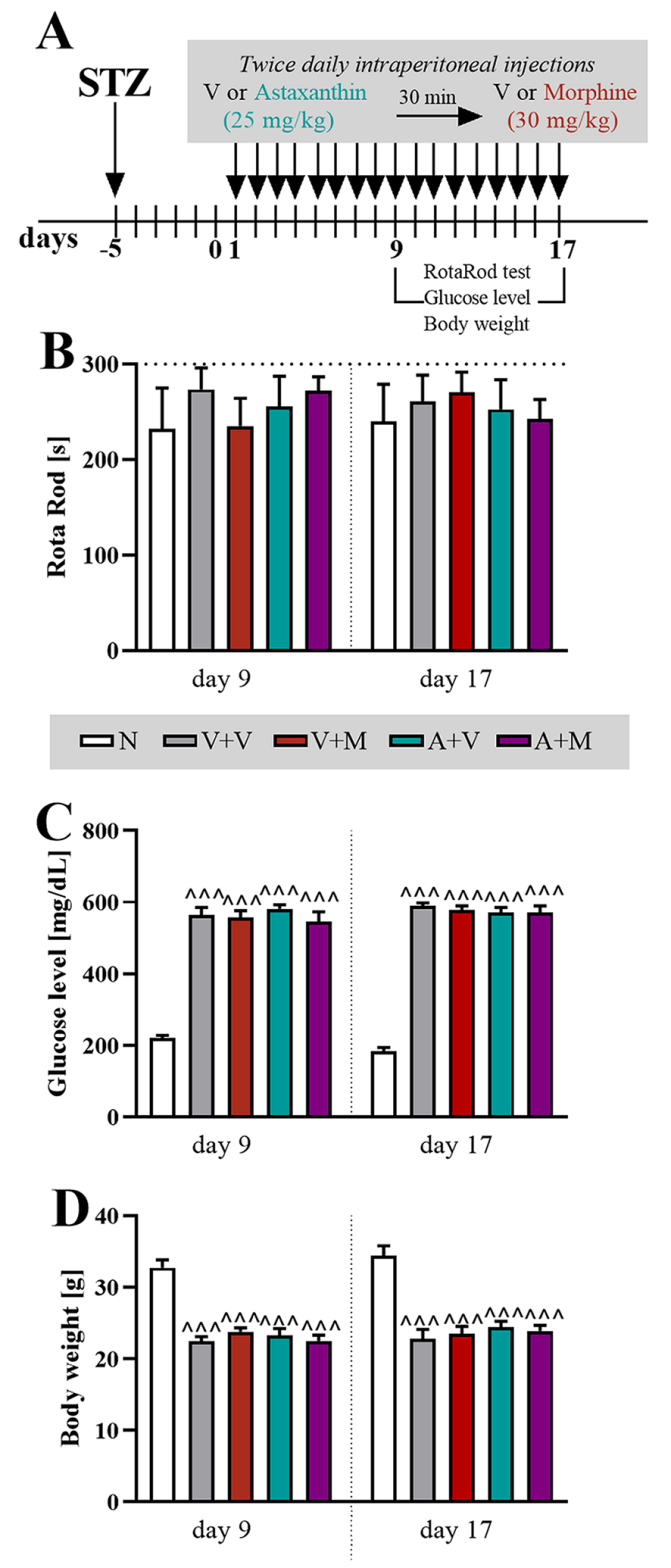
The effects of repeated *ip* administration of astaxanthin (25 mg/kg) or morphine (30 mg/kg) and their coadministration on motor coordination, blood glucose levels and body weight in STZ-treated male mice (**A**). This evaluation was performed on Days 9 and 17 as part of the repeated administration study. The rotarod test was conducted 1 h after the morphine administration (**B**), and glucose level (**C**) and body weight (**D**) measurements were also performed and the numbers of animals are as follows: *N* = 7; V + V = 9; V + M = 9; A + V = 8; A + M = 8. The results were analyzed using one-way ANOVA with Bonferroni’s multiple comparisons *post hoc* test. ^^^ *p* < 0.001 indicates differences between naive and STZ-exposed mice. The dotted lines indicate the cutoff values for the rotarod test. Abbreviations: A, astaxanthin; M, morphine; N, naive; V, vehicle; STZ, streptozotocin

The effect of astaxanthin and morphine on motor coordination in STZ-treated mice was evaluated on days 9 and 17 as a part of the repeated administration study (Fig. 5A). In both this time points the analgesic effects of astaxanthin were observed, however morphine had already lost its effectiveness (Fig. 3B, C). Importantly, the obtained results showed that there was no significant differences in locomotor function among the tested groups (Fig. 5B). On the same days, glycemia and body weight were also measured. All STZ-treated groups of mice had elevated blood glucose levels in comparison to those of naive animals, which was noted at each time point (Fig. 5C). In the case of body weight measurements, animals treated with STZ weighed less than naive mice (Fig. 5D).

## Discussion

The experiments performed in the following behavioral study are the first to demonstrate that in a diabetic neuropathic pain model, both the *it* and *ip* administration of astaxanthin diminishes tactile and thermal hypersensitivity. Moreover, astaxanthin, regardless of the route of administration, exerts analgesic effects in a similar way in male and female mice. These results also provide evidence that systemic, repeated administration of astaxanthin slightly delays the development of morphine tolerance but significantly suppresses the occurrence of OIH, although it does not affect blood glucose levels, body weight or motor coordination. Unexpectedly, astaxanthin administered repeatedly produced a better analgesic effect when administered alone than when it was administered in combination with morphine, as was noted particularly in the von Frey test, and its potency became even more pronounced over time. These results provide a basis for further evaluation of the potential use of astaxanthin in the clinical treatment of diabetic neuropathy and suggest that the multidirectional action of this substance may have positive effects on relieving pain.

This study was performed in a well-established animal model of diabetic neuropathy induced by a single *ip* injection of STZ. The first symptoms of pain appear around day 3, and hypersensitivity is most severe starting on day 6 in both sexes and remains at this level for several weeks [6, 7, 61–66]. In this model, as well as in patients suffering from diabetes, neuropathy develops in sensory and motor nerves [67–69]. In STZ-induced diabetes, increased glucose concentrations in the blood correlate with tactile and thermal hypersensitivity development [27], which is consistent with our results.

In recent years, clinical [70] and experimental [6, 27, 66, 71] research has indicated that the treatment of diabetic neuropathy remains challenging due to the complicated molecular mechanisms. Several studies, including ours, have suggested that MAPKs are involved in hypersensitivity [25–27, 31, 72]. Importantly, the influence of glucolipotoxicity on apoptotic p38 and JNK1/2 signaling has already been proven [73]. Moreover, high glucose levels through ERK1/2 signaling promote defects in insulin secretion [74–77]. It was also reported that in diabetic C57BL/KsJ-db/db mice, astaxanthin exerts beneficial effects by promoting the ability of islet cells to secrete insulin, which results in lower levels of blood glucose [78]. However, the Bhattarai group showed that in STZ-induced diabetic mice, astaxanthin does not directly improve hyperglycemia despite its long-term administration [79]. Similar results were also obtained in STZ-induced diabetic rats, where astaxanthin had no significant effect on blood glucose levels but inhibited oxidative stress and inflammatory mediators in ocular tissues [80]. In agreement with these reports, in our study, we did not observe an influence of astaxanthin on blood glucose levels at any studied time point. However, our research demonstrated that astaxanthin effectively reduced STZ-induced neuropathic pain symptoms. This observation leads to the consideration of which mechanisms of action of this substance may be involved in these beneficial effects. Astaxanthin (3,3′-dihydroxy-β, β′-carotene-4,4′-dione; molecular formula C_40_H_52_O_4_) has been previously reported to have therapeutic antidiabetic, anti-inflammatory, and neuroprotective effects [81]. It is a substance widely found in microalgae, yeast, bacteria and plants, and its chemical and safety profile is well-described [82]. The great advantage of this compound is the fact that thanks to its unique molecular structure, astaxanthin easily crosses the blood–brain barrier [83]. The *ip* administration of astaxanthin relieves neuropathic pain by inhibiting ERK1/2 and p38 activation after spinal nerve ligation in mice [36]. ERK1/2 signaling is linked to potassium channel phosphorylation and changes in voltage-gated calcium channels in sensory neurons [84]. A series of pharmacological studies have shown that selective inhibitors of ERK1/2 (U0126, PD98059, PD198306) [23, 31, 85–87], p38 (SB203580, FR167653) [20, 88–90] and JNK (D-JNKI-1JNK, SP600125) [91, 92] reduce hypersensitivity in different neuropathic pain models. Notably, the first tests were undertaken to evaluate the analgesic potency of the p38 inhibitors (SB-681323, GW856553) in patients suffering from neuropathic pain [93, 94]; however, further evaluation in larger clinical trials is needed. Nevertheless, since p38, ERK1/2 and JNK1/2 inhibitors have been shown to induce pain relief in neuropathic pain models, we assumed that substances acting on the entire MAPK family could be even more effective. One such drug is minocycline, which, in addition to being a p38 inhibitor, also impairs ERK1/2 and JNK signaling [95, 96]. Since astaxanthin is also considered to be a MAPKs inhibitor [33–36], we suspect that these mechanisms, among others, might be responsible for the reduction in hypersensitivity in the mice in our experiments.

Importantly, in 2023, we showed that *it* administration of astaxanthin significantly diminished hypersensitivity in male mice exposed to sciatic nerve injury, and a single administration of morphine evoked better analgesic effects than either of these drugs alone [37]. In the present study, we showed that both *it* and *ip* administration of astaxanthin relieve pain in a STZ-induced neuropathic pain model and, importantly, to a similar extent in both males and females. Additionally, in our opinion, the beneficial effects of astaxanthin are probably not only due to the inhibition of the MAPK family, but also to the fact that this substance is an activator of Nrf2 [97]. Recently, it was shown that Nrf2 is involved in relieving neuropathic pain by reducing neuroinflammation and mitochondrial dysfunction in rodent models [98–100]. Moreover, in 2023, we showed that bardoxolone methyl (Nrf2 activator [101, 102]) diminished tactile and thermal hypersensitivity and enhanced opioid effectiveness in mice exposed to sciatic nerve injury [37]. These data correspond well with the results of other studies, which also revealed the beneficial effects of bardoxolone methyl in ischemic optic [103] and diabetic [104] neuropathy. In summary, we have shown for the first time that a single, systemic administration of astaxanthin has beneficial effects in an STZ-induced diabetic neuropathic pain model in male and female mice. Taking into account the previous studies [82], the doses of astaxanthin we used are non-toxic, and the observations of the mice and behavioral tests revealed no visible adverse effects of astaxanthin, including no impact on the animals’ motor coordination. It should be emphasized that previous research indicate several mechanisms of action of astaxanthin in pain, however, in order to assess whether a peripheral or central mechanisms are responsible for analgesic effect of this substance, more detailed biochemical studies are necessary.

Despite the fact that multiple guidelines for the treatment of diabetic neuropathic pain are available, many drugs do not provide satisfactory pain relief and have side effects that limit their usefulness. It is well known that neuropathic pain and opioid tolerance share common features of the loss of analgesic efficacy of these drugs, which suggests that similar mechanisms are involved in these processes [72, 105–109]. However, further experiments, including pharmacological studies, are needed to explain the cause of the decrease in the effectiveness of morphine in treating neuropathic pain. Some studies indicate that one of the reasons may be highly activated MAPK signaling. Our group reported that repeated systemic administration of minocycline reduced the development of morphine tolerance in a nerve injury-evoked neuropathic pain model [72] because of its ability to inhibit spinal p38 activation, as shown by others [110]. Since then, there has been increasing evidence that MAPKs contribute to the decreased effectiveness of opioids in neuropathy. Moreover, it has recently been demonstrated that Nrf2 activators, such as 5-fluoro-2-oxindole [111] and sulforaphane [112] also enhance morphine analgesia. Therefore, bearing in mind that the inhibition of p38 [108], ERK1/2 [23, 31] and JNK [113, 114] or activation of Nrf2 [111, 112] improves morphine analgesia in neuropathic pain models [27, 108], astaxanthin seemed to be a promising substance, due to its pleiotropic mechanism of action which include modulation of above-mentioned factors. In the current experiments we observed that up to day 7 administration of astaxanthin with morphine evokes pain relief, however in this group we also reported the development of morphine tolerance in both behavioral tests, but importantly astaxanthin prevents the occurrence of OIH, phenomenon which is well described in the literature [115, 116]. This is a state of hypersensitivity to painful stimuli resulting in the exacerbation of a pain sensation [115]. The long-term use of morphine leads to adaptations within downstream signaling pathways of opioid receptors. In tolerance, continued β-arrestin binding to the receptor leads to internalization, degradation, and a decreased number of membrane receptors, while in OIH, the role of factors responsible for reducing antinociceptive systems and amplifying pronociceptive signals has been implicated [116]. Additionally, one of the mechanisms reported to be responsible for the occurrence of OIH is increased activation of N-methyl-D-aspartate (NMDA) receptors, which causes spinal neuron sensitization. It has been reported that the NMDA receptor antagonist MK801 ameliorates opioid-associated hyperalgesia [117, 118]. Recently, molecular docking studies have shown that astaxanthin fits into the inhibitory binding pocket of the NMDA receptor, and it was also reported that this substance beneficially regulates ionotropic glutamate receptors, which prevents excitotoxicity [39, 119]. The results of our experiments clearly indicate that astaxanthin prevents the development of OIH, which may also be related to these effects on NMDA receptors. Unexpectedly, astaxanthin administered alone has better analgesic effects than morphine and it does not lose its effects over time in terms of relieving tactile hypersensitivity. Therefore, it seems that this compound probably has a greater impact on nociceptive transmission through the Aβ fibers responsible for touch than through the C fibers, which conduct thermal stimuli [120]. It was previously showed that in diabetic neuropathic pain model the mechanical hypersensitivity in rats was reversed by acute and repeated administration of p38 inhibitor (SD-282), but this substance prevented the exacerbation of C-, but not Aδ mediated thermal hypersensitivity [121]. However in case of our experiments it needs to be explained in future studies if astaxanthin indeed affects fibers differently. In sum, our results provide a basis for further evaluation of the potential use of astaxanthin in the clinical treatment of diabetic neuropathy alone and as a part of combined therapy.

## Summary

Considering that naturally derived products with pharmacological effects are of great interest to the medical industry in the 21st century, the use of astaxanthin, which has multiple mechanisms of action, may be an important pharmacological tool for the treatment of neuropathic pain, which is a serious and common complication of diabetes. In light of our current behavioral results obtained in a mouse model, we can only hypothesize that inhibition of MAPK signaling, activation of Nrf2 and/or blockade of NMDA receptors play a role in astaxanthin-induced analgesia in diabetic neuropathy. Undoubtedly, further long-term behavioral and molecular studies are needed to explain the neuroimmune background of analgesic action of astaxanthin in diabetes in female and male mice. Nevertheless, taking into account the already known multidirectional mechanism of action of this substance, explaining its long-term analgesia in diabetic neuropathy requires the use of specialized methods with a broad spectrum of analysis. From a clinical point of view, it is worth emphasizing that in our experiments, we noticed not only a reduction in thermal and tactile hypersensitivity but also no side effects in the animals treated with astaxanthin. Moreover, astaxanthin is already used and approved as a dietary supplement, safe for human consumption [122]. Overall, these promising results are important for the development of new, more effective therapeutic interventions; however, continuation of this research is essential to broaden our knowledge regarding the mechanisms of diabetic neuropathy.

## Data Availability

No datasets were generated or analysed during the current study.

## Abbreviations

A: Astaxanthin
ANOVA: Analysis of variance
AUC: Area under curve
DMSO: Dimethyl sulfoxide
ED: Effective dose
ERK: Extracellular-regulated kinase
*ip*: Intraperitoneal
*it*: Intrathecal
JNK: C-Jun N-terminal – kinase
M: Morphine
MAPK: Mitogen-activated protein kinases
N: Naive
NMDA: N-methyl-D-aspartate
Nrf2: Nuclear factor erythroid 2-related factor 2
OIH: Opioid-induced hyperalgesia
SEM: Standard error of the mean
STZ: Streptozotocin
V: Vehicle
